# The current status, knowledge, attitudes, and challenges of generative artificial intelligence use among undergraduate nursing students: a single-center cross-sectional survey of western China

**DOI:** 10.3389/fpubh.2025.1648416

**Published:** 2025-09-16

**Authors:** Yuanyuan Zhao, You Yuan, Zhuosi Wen, Lanlan Leng, Lei Shi, Xinyang Hu, Xiaoman Wei, Meng Zuo, Jianghong Mou, Qian Luo, Mei Chen, Rujun Hu, Huiming Gao

**Affiliations:** ^1^Department of Critical Care Medicine, Affiliated Hospital of Zunyi Medical University, Zunyi, Guizhou, China; ^2^Department of Nursing, Affiliated Hospital of Zunyi Medical University, Zunyi, Guizhou, China; ^3^School of Nursing, Zunyi Medical University, Zunyi, Guizhou, China; ^4^Department of Nursing, Zunyi Medical and Pharmaceutical College, Zunyi, Guizhou, China

**Keywords:** generative artificial intelligence, nursing education, undergraduate nursing students, attitudes, knowledge, challenges

## Abstract

**Background:**

Generative artificial intelligence (Gen AI) is rapidly permeating the fields of education and healthcare, with increasing impact on nursing education. Understanding nursing students’ acceptance of Gen AI and the challenges they face is essential for optimizing future curriculum design.

**Objective:**

This study aimed to assess the current usage, knowledge level, attitudes, and perceived challenges of Gen AI among undergraduate nursing students in western China, to inform the effective integration of AI into nursing education.

**Methods:**

A single-center, cross-sectional study was conducted using a structured, validated questionnaire that covered five domains: demographics, AI tool usage, knowledge, attitude, and challenges. Participants were undergraduate nursing students from Zunyi Medical University. Data were collected via an online platform from May to June 2025 and analyzed using SPSS 29.0 for descriptive and inferential statistics based on demographic subgroups.

**Results:**

A total of 534 valid responses were analyzed. Females accounted for 80.15%, with a mean age of 20.88 years. Grade distribution: sophomore (30.71%), freshman (22.47%), senior (24.53%), and junior (22.28%); 64.79% of students were from urban backgrounds. About 57.86% reported frequent or consistent use of Gen AI tools, mainly via smartphones (94.76%). Most students used 2–3 tools (70.41%), with DeepSeek (72.10%) and Doubao (69.85%) being the most popular. Primary uses included problem-solving (84.46%), course support (66.29%), and academic writing (51.87%). Daily multiple usage was reported by 25.47, and 87.45% used AI for less than 30 min per session. Primary information sources were social media (78.09%) and peer recommendations (71.35%). Median scores: knowledge 3.43 (IQR 2.86–3.86), attitude 3.58 (IQR 3.33–3.83), challenges 3.50 (IQR 3.17–3.92). Only 38.01% received AI-related training; 83.33% found it challenging to ask probing or insightful questions when using Gen AI. Students demonstrated moderate knowledge and positive attitudes, but faced notable concerns, particularly regarding data privacy, tool reliability, and the impact on critical thinking skills.

**Conclusion:**

Undergraduate nursing students in western China exhibit a generally positive yet cautious attitude toward Gen AI. Targeted educational interventions are recommended to address their concerns and enhance the benefits of AI in nursing education. Future research should focus on the development of AI literacy and the long-term implications of integrating AI into clinical nursing practice.

## 1 Introduction

Artificial Intelligence (AI) is an interdisciplinary technological system that relies on data as a key resource. By leveraging machine learning algorithms to simulate human cognitive functions, AI is capable of performing tasks such as language processing, learning, problem-solving, and autonomous decision-making in complex environments, thereby providing strong support and assistance to humans (1, 2). Within the broader field of AI research, Generative artificial intelligence (Gen AI) constitutes a critical subset, focusing on the creative or human-like generation of new content, including text, images, audio, video, code, multimodal integration, and other data formats (3, 4).

Since the launch of the GPT series in 2018, and especially after ChatGPT gained global attention in 2022, Gen AI has rapidly expanded across industrial and academic domains (5, 6). Representative international models include GPT-4, DALL·E 3, Sora, Gemini, Claude, Grok-3, LLaMA 3, Stable Diffusion XL, Midjourney, and Runway. At the same time, domestic models such as DeepSeek, ModelScope, ERNIE, Spark, Kimi, BaiChuan, ZhiPu AI, and DouBao have also been developing rapidly in China, promoting global technological and ecosystem integration.

However, the widespread use of Gen AI also brings a range of challenges, including students’ overreliance on AI, which can lead to a decline in autonomous learning ability. Additionally, there are risks of bias and inaccuracies in generated content, concerns over academic integrity, including plagiarism, as well as data privacy and ethical issues (7–11). These challenges are particularly critical in nursing education, as nursing students are in a key stage of developing clinical thinking and professional skills. Their knowledge and acceptance of AI not only influence their current learning approaches but will also shape future patterns of human-AI collaboration in clinical practice (12). Therefore, accurately assessing nursing students’ current understanding of AI is crucial for developing targeted AI training programs and optimizing future nursing talent cultivation.

Existing studies indicate significant regional differences in students’ knowledge, attitudes, and usage of AI (13–15). For example, students in Jordan possess limited AI knowledge and exhibit cautious attitudes, facing barriers in practical application (16); nursing students in Pakistan mostly lack AI experience, learn about AI primarily through social media, and are most concerned about data privacy (17); Egyptian students demonstrate moderate knowledge (61%) of Gen AI and positive attitudes (47%) but limited application (18); in China, students have low levels of AI knowledge (only 38.3% understand it) but show strong willingness to use it (50% support) (19); Saudi students’ attitudes towards AI are influenced by personality traits, with openness correlating to positive views. Primary concerns include cost, lack of training, and system reliability (20); and American nursing students maintain a cautiously optimistic view of Gen AI outputs, stressing the need for result verification (14). Despite these insights into the diversity of students’ AI-related perceptions and practices across different regions, there is a lack of research focusing on undergraduate nursing students in western China (21–23). Most existing studies primarily focus on qualitative exploration and lack in-depth questionnaire surveys on the current status of AI usage. There is a need for more quantitative studies that substantiate the benefits and limitations that students encounter with AI usage (24).

In light of the limitations of existing research and the practical needs of the field, this study focuses on undergraduate nursing students in economically underdeveloped regions of western China, using students from Zunyi Medical University as the sample. As a representative medical education institution in the West, the university’s nursing students bring unique educational and practical backgrounds, providing a solid empirical foundation for the study. Through a structured questionnaire survey, this research systematically assessed students’ knowledge, usage, attitudes, and challenges related to Gen AI. The findings aim to provide valuable insights for nursing educators, supporting the effective integration of AI into nursing education by balancing technological innovation with risk management and optimizing instructional strategies to promote the deep integration of AI and nursing education.

## 2 Methods

### 2.1 Study design

This study employed a single-center, cross-sectional design and surveyed undergraduate nursing students at Zunyi Medical University between May and June 2025. The student body hails predominantly from the western provinces and municipalities of Yunnan, Guizhou, Sichuan, Chongqing, Guangxi, and Shaanxi, with a small proportion from other regions; their urban–rural and gender distributions closely mirror those of peer medical universities in western China, making the sample representative of the overall characteristics of nursing students in underdeveloped western areas. Adhering to the STROBE guidelines, we used a structured questionnaire to quantitatively assess the current use, knowledge, attitudes, and perceived challenges of Gen AI (25).

### 2.2 Design and preliminary validation of questionnaire

This study aimed to investigate undergraduate nursing students’ knowledge, usage, attitudes, and perceived challenges regarding generative AI. To achieve this, the Generative AI Literacy Questionnaire for Nursing Students (GAILQ-NS) was developed, drawing on prior studies in Pakistan and recent progress in nursing and AI (19, 26).

**Figure 1:**
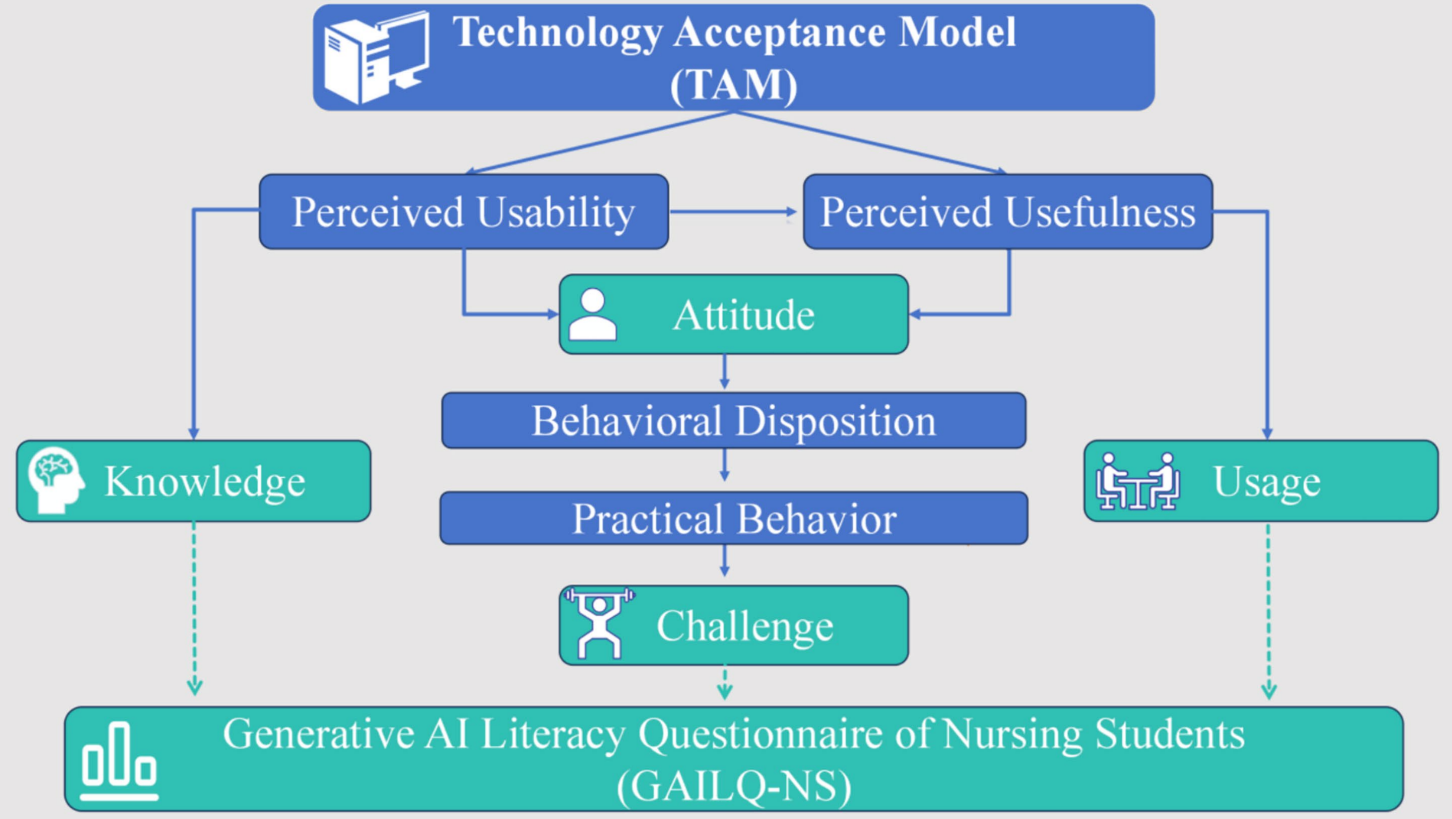
TAM-based framework diagram of GAILQ-NS. Illustrating the GAILQ-NS framework based on TAM, connecting perceived usability and perceived usefulness to knowledge, usage, attitude, and challenges, with practical behavior mediating between behavioral disposition and usage.

The Technology Acceptance Model (TAM) served as the theoretical framework, highlighting perceived usefulness, perceived ease of use, attitude, and behavioral intention (27). Guided by TAM, the GAILQ-NS was structured into four dimensions: knowledge, usage, attitude, and perceived challenges. Knowledge reflects students’ AI literacy and informs their perceived usefulness and ease of use; usage assesses actual engagement; attitude corresponds to the TAM’s construct of user attitude; and perceived challenges capture the barriers encountered when translating behavioral intention into practice, thereby identifying the difficulties students face in adopting generative AI. This theoretical alignment provided the questionnaire with both a solid foundation and a coherent structure. The TAM-based framework of GAILQ-NS is shown in Figure 1.

The draft questionnaire was reviewed by five nursing education experts (PhDs) and two AI specialists, and revised through three iterative rounds of feedback. Content validity was subsequently evaluated by 24 experts, using the Content Validity Index (CVI). A four-point scale (1 = not relevant, 2 = somewhat relevant, 3 = quite relevant, 4 = highly relevant) was applied, with Item-level CVI (I-CVI) calculated as the proportion of experts rating an item as 3 or 4. Results showed I-CVI values of 0.905 (usage), 0.874 (knowledge), 0.857 (attitude), and 0.919 (challenges), with an overall Scale-level CVI (S-CVI) of 0.91, confirming high content validity.

A pilot test with 50 nursing students further demonstrated the questionnaire’s feasibility, clarity, and reliability, with an average completion time of 3 min. Internal consistency, assessed using Cronbach’s alpha, yielded coefficients of 0.669 (usage), 0.802 (knowledge), 0.914 (attitude), and 0.919 (challenges), indicating satisfactory to excellent reliability. These procedures collectively ensured the scientific rigor and practical utility of the GAILQ-NS for broader application in nursing education and research.

### 2.3 Study population and inclusion/exclusion criteria

Ethics approval was obtained from the Biomedical Research Ethics Committee of the Affiliated Hospital of Zunyi Medical University (KLL-2025-069). Using a convenience sampling approach, we distributed the GAILQ-NS to 2,340 undergraduate nursing students enrolled at Zunyi Medical University. Inclusion criteria for the questionnaire were: currently enrolled in the nursing undergraduate program and willing to participate. Students on leave, in military service, or unwilling to join were excluded from the program. Responses that were incomplete, illogical, or submitted in under 2 min were also excluded.

### 2.4 Sample size

This study employed a cross-sectional design to evaluate nursing students’ use, attitudes, and challenges related to Gen AI. The questionnaire included 39 items; following the rule of 5–10 times the number of items, the maximum sample size was 390. Using the Calculator.net sample size calculator (with a 5% margin of error, 95% confidence level, and 50% proportion),^1^ the estimated sample size was 385. Considering these results and a 10% invalid response rate, the final sample size was set at 429 to ensure accuracy and reliability.

### 2.5 Questionnaire content

The GAILQ-NS consists of five parts. Apart from the first section, which collects sociodemographic information including gender, age, grade, and place of residence, a total of 39 questions were designed. The second part, “Current Use of Gen AI,” comprises eight questions, including four single-choice and four multiple-choice questions. These questions cover AI usage frequency, installation locations, quantity, commonly used tools, and average duration of use. The third part, “Current Knowledge of Gen AI,” assesses students’ understanding of basic AI concepts through seven questions, including knowledge of AI, machine learning, and deep learning, as well as their applications in medicine and nursing. The fourth part, “Attitudes toward Use,” comprises 12 questions that explore students’ attitudes toward the application of AI in the medical and nursing fields. The fifth part, “Challenges in Use,” contains 12 questions aimed at understanding the difficulties and challenges students face when using Gen AI tools, such as technical operation, data synchronization, generation of personalized nursing plans, cost, rapid technological iteration, data security, and cultural differences.

In the domains of knowledge, attitude, and challenges, we employed a composite scoring approach rather than an item-level scoring method. All questions were rated on a five-point Likert scale, where 1 stands for “Strongly Disagree,” 2 for “Disagree,” 3 for “Neutral,” 4 for “Agree,” and 5 for “Strongly Agree.” Negatively worded items were reverse-scored (i.e., one became 5, 2 became 4, and 3 remained unchanged) to ensure that higher scores consistently reflected more positive responses. For each domain, the composite mean score was calculated by summing the relevant item scores within the domain and dividing by the number of items. In the knowledge domain, total scores ranged from 7 to 35, with higher scores indicating a better understanding of Gen AI concepts and applications. In the attitude domain, scores ranged from 12 to 60, with higher scores reflecting a more positive attitude toward using Gen AI, including recognition of its potential value and willingness to apply it in practice. In the challenges domain, scores also ranged from 12 to 60, with higher scores indicating that students perceived greater difficulties or barriers, such as technical complexity, skill limitations, or concerns related to data and ethics. Furthermore, to facilitate interpretation, composite scores were categorized into three levels based on common practice in Likert-scale research: low (1.00–2.49), moderate (2.50–3.49), and high (3.50–5.00).

### 2.6 GAILQ-NS distribution and data collection

The GAILQ-NS was designed using the Questionnaire Star app, and a QR code was subsequently generated. Teachers distributed the QR code via class groups on the Xuexitong app, which is installed on all enrolled students’ devices. Participants scanned the QR code through WeChat, QQ, or Xuexitong to learn about the study’s purpose and significance. After providing online informed consent, they anonymously completed the GAILQ-NS. The collected data were then exported from Questionnaire Star for analysis.

### 2.7 Data analysis

The GAILQ-NS data were entered and organized using Excel and subsequently imported into SPSS version 29.0 for statistical analysis. Categorical variables were described using frequencies and percentages, while continuous variables were expressed as means and standard deviations. Group comparisons for categorical variables were conducted using the chi-square test, and for continuous variables with non-normal distribution, the Kruskal-Wallis test was applied. Scores related to knowledge, attitudes, and challenges regarding GenAI were presented as medians and interquartile ranges, with group differences analyzed using the Mann–Whitney U test or Kruskal-Wallis test. Negatively worded items within the attitude dimension were reverse-coded to ensure consistency. Statistical significance was set at an alpha level of 0.05, with *p*-values of less than 0.05 considered statistically significant.

### 2.8 Quality control

The GAILQ-NS was created and distributed via the “Questionnaire Star” platform. The purpose of the study and the principles of completion, including the informed consent principle, were clearly stated at the beginning of the GAILQ-NS. All questions were set as mandatory, and automatic validation was used to prevent duplicate submissions. The GAILQ-NS was completed anonymously, and contact information was collected only from participants who voluntarily agreed to participate in qualitative interviews. After collection, two researchers reviewed the responses and excluded those that were logically inconsistent, had an excessively short completion time, or contained abnormal answers to ensure data quality.

## 3 Results

### 3.1 Demographic characteristics

**Figure 2:**
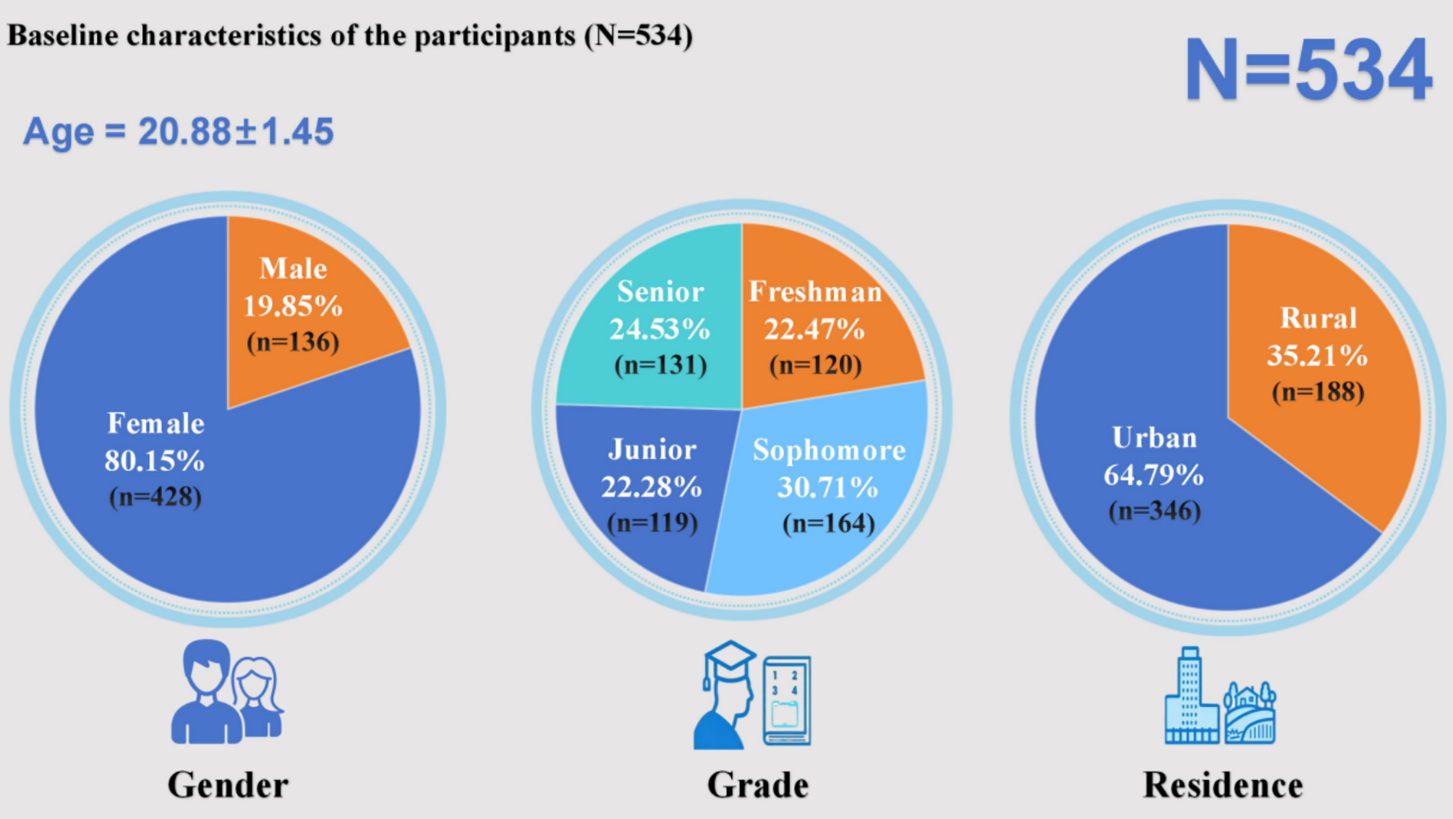
Baseline characteristics of the participants (*N* = 534).

A total of 567 questionnaires were collected in this survey. After excluding 15 responses completed in under 2 min and 18 with identical answers, 534 valid responses were retained, resulting in an effective response rate of approximately 94.19%. As shown in Figure 2, the sample was predominantly female (80.15%), with an average age of 20.88 years (SD = 1.45). In terms of academic level, the highest proportion of participants was sophomores (30.71%), followed by freshmen (22.47%), seniors (24.53%), and juniors (22.28%). Regarding place of residence, students from urban areas accounted for 64.79%, significantly higher than those from rural areas (35.21%). These findings highlight the demographic characteristics of the nursing student population, particularly the predominance of female students and urban backgrounds.

### 3.2 Current status of Gen AI tool usage

Survey results (Table 1) indicate that Gen AI tools have gained substantial traction among undergraduate nursing students. In terms of Overall Usage, 20.22% of respondents reported “Always” using such tools, while 37.64% selected “Often,” totaling 57.86% of high-frequency users. Only 1.5% indicated they had “Never” used AI tools. Regarding installation platforms, mobile phones were the dominant medium, with 94.76% of students accessing AI tools via smartphones, far exceeding usage on Computers (36.14%) and Tablets (24.53%). This highlights not only the prevalence of mobile learning habits but also the deep integration of Gen AI into students’ daily academic and personal routines.

**Table 1:** Current status of Gen AI tools usage (*N* = 534).

Item	Option	Number (n)	Percentage (%)
Overall usage	Always	108	20.22%
	Often	201	37.64%
	Sometimes	167	31.27%
	Occasionally	50	9.36%
	Never	8	1.50%
^*^Installation locations of tools	Mobile phone	506	94.76%
	Computer	193	36.14%
	Tablet	131	24.53%
	Other	12	2.25%
Number of AI tools utilized	One	92	17.23%
	Two to three	376	70.41%
	Four or more	66	12.36%
^*^Most used AI tools	DeepSeek	385	72.10%
	Doubao	373	69.85%
	Quark	322	60.30%
	UniSearch	206	38.58%
	Kimi	96	17.98%
	ChatGPT	40	7.49%
	Other	195	36.52%
Frequency of AI tools use	Multiple times per day	136	25.47%
	Once per day	82	15.36%
	3–5 times per week	194	36.33%
	1–2 times per week	88	16.48%
	1–3 times per month	34	6.37%
Average duration of use	<15 min	227	42.51%
	15–30 min	240	44.94%
	30–60 min	48	8.99%
	>60 min	19	3.56%
^*^AI tools functions	Problem-solving	451	84.46%
	Course support	354	66.29%
	Academic writing	277	51.87%
	Study planning/time management	256	47.94%
	Explore Interest Areas	243	45.51%
	Language learning	176	32.96%
	Entertainment	47	8.80%
	Other	40	7.49%
^*^Sources of AI tools	Friends/classmates	381	71.35%
	Social media	417	78.09%
	School	175	32.77%
	App store	166	31.09%
	Ads	30	5.62%

* Multiple-choice questions.

In terms of usage patterns, students predominantly adopted a multi-tool strategy. According to the Number of AI Tools Utilized, 70.41% reported using “Two to three” AI tools, while only 17.23% used “One.” Preferences for specific tools revealed a strong inclination toward domestic platforms. DeepSeek (72.10%) and Doubao (69.85%) had significantly higher adoption rates compared to international tools such as ChatGPT (7.49%). Additionally, Quark (60.30%) and Uni-Search (38.5%) also demonstrated substantial user bases. As shown in AI Tools Functions, the usage was highly academic-oriented: 84.46% used AI tools for “Problem-solving,” 66.29% for “Course support,” and 51.87% for “Academic writing.” 47.94% for “Study planning/time management,” and 45.51% for “Explore Interest Areas.” In contrast, only 8.8% utilized them for “Entertainment,” indicating a clear emphasis on the functional and academic value of AI tools among students.

Further analysis of usage behaviors revealed a pattern of frequent and fragmented interaction with AI tools. In the Frequency of AI Tools Use category, 25.47% of students reported using AI tools “Multiple times per day,” and 36.33% used them “3–5 times per week.” Regarding the Average Duration of Use, 87.45% of users kept their sessions under 30 min, with 42.51% reporting use of “<15 min” and 44.94% within “15–30 min.” As for the Sources of AI Tools, social media (78.09%) and peer friends/classmates (71.35%) were the primary channels for discovering AI tools, while formal sources such as schools (32.77%) and app stores (31.09%) played a relatively minor role.

These findings suggest that within the nursing education context in western China, Gen AI tools are rapidly spreading through informal networks. However, their integration into the formal institutional education systems of nursing schools remains limited, indicating a need for further development in this area.

### 3.3 Current status on knowledge of Gen AI tools

The survey results (Table 2) reveal varying levels of basic knowledge about AI among respondents. Over 65% of students agreed or strongly agreed that they understand what AI is (Q1). However, knowledge related to machine learning and deep learning was relatively low, with only about 52% of students expressing familiarity (Q2), despite a similar median score of 4 (2, 5), indicating more variation and uncertainty in this area. Regarding the applications of AI in the medical field, nearly 56% reported some degree of familiarity (Q3), while knowledge of AI applications specifically in nursing was slightly lower, at approximately 48% (Q5). Additionally, fewer than 36% of students reported having received any AI-related education or training before the survey (Q4), with a median score of 3 (range: 2–4). This suggests limited access to formal learning opportunities during their undergraduate nursing program.

**Table 2:** Current status on knowledge of Gen AI tools (*N* = 534).

Questions	Strongly disagree (1)	Disagree (2)	Neutral (3)	Agree (4)	Strongly agree (4)	M (Q₁, Q₃)	95% CI
Q1: I know what artificial intelligence is.	13 (2.43%)	11 (2.06%)	161 (30.15%)	159 (29.78%)	190 (35.58%)	4 (3, 5)	3.86–4.02
Q2: I know about machine learning or deep learning.	70 (13.11%)	77 (14.42%)	109 (20.41%)	140 (26.22%)	138 (25.84%)	4 (2, 5)	3.26–3.49
Q3: I am familiar with the applications of artificial intelligence in the medical field.	28 (5.24%)	29 (5.43%)	177 (33.15%)	151 (28.28%)	149 (27.90%)	4 (3, 5)	3.59–3.77
Q4: Have I received any education or training related to artificial intelligence?	116 (21.72%)	121 (22.66%)	106 (19.85%)	97 (18.16%)	94 (17.60%)	3 (2, 4)	2.75–2.99
Q5: I know about the applications of artificial intelligence in the nursing field.	51 (9.55%)	47 (8.80%)	176 (32.96%)	138 (25.84%)	122 (22.85%)	3 (3, 5)	3.33–3.54
Q6: I am familiar with current Gen artificial intelligence.	49 (9.18%)	58 (10.86%)	173 (32.40%)	116 (21.72%)	138 (25.84%)	3 (3, 5)	3.34–3.55
Q7: I can ask practical questions to artificial intelligence.	138 (25.84%)	213 (39.89%)	56 (10.49%)	55 (10.30%)	72 (13.48%)	2 (1, 3)	2.34–2.57

M: Median, Q₁: 1st Quartile, Q₃: 3rd Quartile.

In terms of GenAI (Q6), with a median score of 3 (3, 5), about 47% of students expressed familiarity. However, a significant portion remained neutral or unaware, indicating that this area is still not widely understood. Notably, only about 24% of students believed they could ask practical questions to AI systems (Q7); this item had the lowest median score of 2 (1, 3), indicating generally low confidence and limited skill in practical AI interaction. Most respondents showed low confidence in this skill, which may hinder their efficient use of AI tools. Overall, the findings suggest that students’ understanding of AI is still in its early stages, particularly regarding Gen AI and practical application skills. This highlights the need for enhanced education and training efforts, such as incorporating Gen AI literacy training workshops or courses into the nursing curriculum.

### 3.4 Current status on attitudes of Gen AI tools

The attitude dimension survey results (Table 3) indicate that respondents generally recognize the significant role of AI in the medical field. Over 74% of students (Q1) believe AI is substantial for medical development, and nearly 61% (Q2) believe AI will become a key part of future healthcare systems. Most students also agree that AI can effectively improve the quality and efficiency of clinical nursing (Q3, 73.78% agree or strongly agree) and support the introduction of AI alongside advancements in medical technology (Q4, 71.16% agree or strongly agree). Regarding nursing specifically, about 66.29% consider AI very important (Q5), and 60.67% support allocating budgets for the development of AI in healthcare technology (Q6). Additionally, over 72% of students (Q7) hope AI-related training will be included in nursing school curricula, indicating a strong demand for AI education within the nursing field.

**Table 3:** Current status on attitudes of Gen AI tools (*N* = 534).

Questions	Strongly disagree (1)	Disagree (2)	Neutral (3)	Agree (4)	Strongly agree (4)	M (Q₁, Q₃)	95% CI
Q1: I believe AI plays a vital role in medical development.	7 (1.31%)	9 (1.69%)	120 (22.47%)	259 (48.5%)	139 (26.03%)	4 (3, 5)	3.89–4.03
Q2: I believe AI will become a crucial part of future healthcare systems.	8 (1.5%)	15 (2.81%)	187 (35.02%)	223 (41.76%)	101 (18.91%)	4 (3, 4)	3.67–3.81
Q3: I believe AI can effectively improve the quality and efficiency of clinical nursing.	9 (1.69%)	6 (1.12%)	125 (23.41%)	284 (53.18%)	110 (20.6%)	4 (3, 4)	3.83–3.97
Q4: I think it’s necessary to introduce AI with the advancement of medical technology.	7 (1.31%)	13 (2.43%)	134 (25.09%)	264 (49.44%)	116 (21.72%)	4 (3, 4)	3.81–3.95
Q5: I believe AI is essential in the field of nursing.	10 (1.87%)	12 (2.25%)	158 (29.59%)	251 (47%)	103 (19.29%)	4 (3, 4)	3.72–3.87
Q6: I support allocating budgets for the development of AI in healthcare technology.	10 (1.87%)	20 (3.75%)	180 (33.71%)	233 (43.63%)	91 (17.04%)	4 (3, 4)	3.63–3.77
Q7: I hope AI-related training will be included in medical school curricula.	9 (1.69%)	10 (1.87%)	130 (24.34%)	265 (49.63%)	120 (22.47%)	4 (3, 4)	3.82–3.96
^*^Q8: I worry that AI might replace nurses in the future.	103 (19.29%)	159 (29.78%)	123 (23.03%)	89 (16.67%)	60 (11.24%)	3 (2, 4)	3.18–3.40
^*^Q9: I am anxious about the potential misdiagnosis or technology dependency caused by AI.	18 (3.37%)	55 (10.3%)	250 (46.82%)	149 (27.9%)	62 (11.61%)	3 (2, 3)	2.58–2.74
^*^Q10: I think AI could become a burden for nursing professionals.	22 (4.12%)	93 (17.42%)	189 (35.39%)	160 (29.96%)	70 (13.11%)	3 (2, 3)	2.61–2.78
^*^Q11: I worry that AI may weaken nurses’ ability to provide humanistic care.	112 (20.97%)	192 (35.96%)	142 (26.59%)	56 (10.49%)	32 (5.99%)	4 (3, 4)	3.46–3.65
Q12: I am willing to recommend the use of AI tools in nursing practice.	8 (1.5%)	9 (1.69%)	84 (15.73%)	195 (36.52%)	238 (44.57%)	4 (4, 5)	4.14–4.28

* Reverse Coding: For items Q8 to Q11, negative items will have their scores reversed (1–5 becomes 5–1). M: Median, Q₁: 1st Quartile, Q₃: 3rd Quartile.

However, some students express concerns about the potential negative impacts of AI. Approximately 28% worry that AI may replace nurses in the future (Q8), and 40% feel anxious about the potential for misdiagnoses or overreliance on AI technology (Q9). Around 43.07% agree that AI could become a burden for nursing professionals (Q10). Moreover, nearly 16.48% are concerned that AI might weaken nurses’ ability to provide humanistic care (Q11, agree and strongly agree). Despite these concerns, a large majority (81.09%, Q12) are still willing to recommend the use of AI tools in nursing practice, with the highest observed median score of 4 (4, 5), reflecting an overall positive attitude and acceptance toward AI.

In summary, undergraduate nursing students hold a positive perception of Gen AI technology, acknowledging its application value and development potential, while remaining cautiously aware of the challenges and risks AI may bring. The central tendency measures, particularly the consistent median of 4 across most positive statements, further reinforce this optimistic yet balanced viewpoint.

### 3.5 Current status on challenges of Gen AI tools

The challenge dimension survey results (Table 4) indicate that undergraduate nursing students have specific concerns regarding the application of GenAI (Gen AI) in clinical practice, particularly in terms of technical effectiveness and system usability. A total of 43.45% of students believed that AI struggles to provide effective nursing decisions (Q1), and 43.26% indicated that AI fails to generate personalized care plans based on individual patient needs (Q4). Additionally, 37.27% of students were concerned that nursing data cannot be synchronized with AI systems promptly (Q3). In comparison, 31.27% found the AI interface to be complex (Q2), suggesting that user interaction still requires optimization.

**Table 4:** Current status on challenges of Gen AI tools (*N* = 534).

Questions	Strongly disagree (1)	Disagree (2)	Neutral (3)	Agree (4)	Strongly agree (4)	M (Q₁, Q₃)	95% CI
Q1: I think AI is challenging to provide effective nursing decisions.	11 (2.06%)	48 (8.99%)	243 (45.51%)	170 (31.84%)	62 (11.61%)	3 (3, 4)	3.34–3.49
Q2: I think the AI system’s interface is complex.	16 (3%)	86 (16.1%)	265 (49.63%)	113 (21.16%)	54 (10.11%)	3 (3, 4)	3.11–3.27
Q3: I think nursing data cannot be synchronized with the AI system promptly.	12 (2.25%)	74 (13.86%)	249 (46.63%)	135 (25.28%)	64 (11.99%)	3 (3, 4)	3.23–3.39
Q4: I think AI cannot generate personalized nursing plans based on each patient’s unique needs.	17 (3.18%)	58 (10.86%)	228 (42.7%)	168 (31.46%)	63 (11.8%)	3 (3, 4)	3.30–3.46
Q5: I think the cost of paying for AI is high.	12 (2.25%)	42 (7.87%)	215 (40.26%)	197 (36.89%)	68 (12.73%)	3 (3, 4)	3.42–3.58
Q6: I think AI technology evolves quickly and becomes obsolete easily.	13 (2.43%)	55 (10.3%)	230 (43.07%)	183 (34.27%)	53 (9.93%)	3 (3, 4)	3.31–3.47
Q7: I am concerned that AI analyzing personal data may lead to data leakage.	11 (2.06%)	11 (2.06%)	167 (31.27%)	258 (48.31%)	87 (16.29%)	4 (3, 4)	3.68–3.82
Q8: I am concerned about the copyright of data sources retrieved by AI.	12 (2.25%)	12 (2.25%)	182 (34.08%)	246 (46.07%)	82 (15.36%)	4 (3, 4)	3.63–3.77
Q9: I am unclear about who takes the lead in human-AI collaborative nursing.	38 (7.12%)	101 (18.91%)	192 (35.96%)	153 (28.65%)	50 (9.36%)	3 (2, 4)	3.05–3.23
Q10: I think AI in nursing does not fully consider cultural differences.	15 (2.81%)	58 (10.86%)	215 (40.26%)	185 (34.64%)	61 (11.42%)	3 (3, 4)	3.33–3.49
Q11: I find it challenging to ask valuable (probing or insightful) questions when interacting with AI.	2 (0.37%)	9 (1.69%)	78 (14.61%)	182 (34.08%)	263 (49.25%)	4 (4, 5)	4.23–4.37
Q12: I am concerned that long-term dependence on AI will reduce critical thinking skills.	12 (2.25%)	30 (5.62%)	154 (28.84%)	231 (43.26%)	107 (20.04%)	4 (3, 4)	3.65–3.81

M: Median, Q₁: 1st Quartile, Q₃: 3rd Quartile.

In terms of cost and technological change, 49.62% of students felt that the cost of using AI was high (Q5), and 44.2% worried that rapid AI updates might render tools quickly obsolete (Q6). Meanwhile, data security and ethical issues emerged as major concerns—64.6% of students were worried about potential data breaches when AI analyzes personal information (Q7), and 61.43% were concerned about the copyright legality of data used by AI systems (Q8). These findings reveal that while students are aware of AI’s benefits, they remain highly cautious of the associated risks.

Regarding human-AI collaboration and cognitive impact, 38.01% of students expressed uncertainty about who should take the lead in collaborative nursing with AI (Q9), and nearly 46.06% believed that AI fails to adequately consider cultural differences in care (Q10), raising concerns about its adaptability in diverse nursing contexts. Notably, as many as 83.33% of students reported difficulty in asking probing or insightful questions when interacting with AI (Q11), with the highest median score of 4 (4, 5), highlighting that users’ ability to pose practical questions to AI remains a significant challenge. Additionally, 63.3% were concerned that long-term reliance on AI could weaken their critical thinking skills (Q12), posing challenges to their professional development and clinical judgment.

In summary, nursing undergraduates have a clear understanding of the challenges posed by Gen AI, particularly in terms of system usability, data security, personalized service, human-AI role distribution, and the potential impact on professional competencies. The median values (M) for most challenge items range from 3 to 4, suggesting a moderate to high level of concern among respondents. Notably, the median for Q11 (“I find it challenging to ask valuable (probing or insightful) questions when interacting with AI”) reached 4, highlighting a particularly prominent communication barrier. At the same time, other items such as Q1, Q4, and Q12 also recorded medians of 3 or above, indicating widespread recognition of technical and cognitive challenges. Future efforts should focus on optimizing AI systems and enhancing training to improve usability and foster trust in clinical settings.

### 3.6 Gen AI tool use, knowledge, attitudes, challenges, and group differences

To examine differences in GenAI tool use by gender, grade level, and place of residence, this study employed chi-square (*χ*^2^) tests to analyze the distribution of categorical variables, with statistical significance set at *p* < 0.05. Results (Table 5) showed no significant differences between the gender groups in overall usage frequency (*χ*^2^ = 3.14, *p* = 0.535), the number of tools used (*χ*^2^ = 0.81, *p* = 0.667), frequency of use (*χ*^2^ = 1.27, *p* = 0.866), or average duration of use (*χ*^2^ = 3.20, *p* = 0.362). Among grade groups, there was a significant difference in overall usage frequency (*χ*^2^ = 24.82, *p* = 0.016), with first- and second-year students having higher proportions of “always” and “often” use. In contrast, seniors showed a lower continuous usage rate. Differences in the number of tools used (*χ*^2^ = 3.41, *p* = 0.756), frequency of use (*χ*^2^ = 56.24, *p* < 0.001), and average duration of use (*χ*^2^ = 9.43, *p* = 0.398) were also observed for some indicators, with sophomores exhibiting the highest and most significant rate of multiple daily uses. Regarding place of residence, overall usage frequency showed a marginally significant difference (*χ*^2^ = 9.37, *p* = 0.052), and the number of tools used differed significantly (*χ*^2^ = 8.24, *p* = 0.016), with rural students using a more varied range of tools than urban students. However, differences in usage frequency and average duration were not significant.

**Table 5:** Differences in Gen AI tools usage by gender, grade, and residence (*N* = 534).

Variables	Total (*n* = 534)	Male (*n* = 106)	Female (*n* = 428)	Statistic	*p*	Freshman (*n* = 120)	Sophomore (*n* = 164)	Junior (*n* = 119)	Senior (*n* = 131)	Statistic	*p*	Rural (*n* = 188)	Urban (*n* = 346)	Statistic	*p*
Overall usage				*χ*^2^ = 3.14	0.535					*χ*^2^ = 24.82	**0.016**			*χ*^2^ = 9.37	0.052
Always	108 (20.22)	24 (22.64)	84 (19.63)			32 (26.67)	34 (20.73)	24 (20.17)	18 (13.74)			51 (27.13)	57 (16.47)		
Often	201 (37.64)	36 (33.96)	165 (38.55)			48 (40.00)	67 (40.85)	47 (39.50)	39 (29.77)			68 (36.17)	133 (38.44)		
Sometimes	167 (31.27)	31 (29.25)	136 (31.78)			29 (24.17)	51 (31.10)	35 (29.41)	52 (39.69)			52 (27.66)	115 (33.24)		
Occasionally	50 (9.36)	12 (11.32)	38 (8.88)			7 (5.83)	11 (6.71)	12 (10.08)	20 (15.27)			14 (7.45)	36 (10.40)		
Never	8 (1.50)	3 (2.83)	5 (1.17)			4 (3.33)	1 (0.61)	1 (0.84)	2 (1.53)			3 (1.60)	5 (1.45)		
Number of AI tools utilized				*χ*^2^ = 0.81	0.667					*χ*^2^ = 3.41	0.756			*χ*^2^ = 8.24	**0.016**
One	92 (17.23)	21 (19.81)	71 (16.59)			19 (15.83)	24 (14.63)	22 (18.49)	27 (20.61)			23 (12.23)	69 (19.94)		
Two to three	376 (70.41)	71 (66.98)	305 (71.26)			84 (70.00)	118 (71.95)	82 (68.91)	92 (70.23)			134 (71.28)	242 (69.94)		
Four or more	66 (12.36)	14 (13.21)	52 (12.15)			17 (14.17)	22 (13.41)	15 (12.61)	12 (9.16)			31 (16.49)	35 (10.12)		
Frequency of AI tool use				*χ*^2^ = 1.27	0.866					*χ*^2^ = 56.24	**<0.001**			*χ*^2^ = 3.04	0.551
Multiple times per day	136 (25.47)	30 (28.30)	106 (24.77)			32 (26.67)	62 (37.80)	27 (22.69)	15 (11.45)			45 (23.94)	91 (26.30)		
Once per day	82 (15.36)	15 (14.15)	67 (15.65)			25 (20.83)	24 (14.63)	19 (15.97)	14 (10.69)			32 (17.02)	50 (14.45)		
3–5 times per week	194 (36.33)	38 (35.85)	156 (36.45)			47 (39.17)	52 (31.71)	40 (33.61)	55 (41.98)			71 (37.77)	123 (35.55)		
1–2 times per week	88 (16.48)	15 (14.15)	73 (17.06)			15 (12.50)	23 (14.02)	20 (16.81)	30 (22.90)			32 (17.02)	56 (16.18)		
1–3 times per month	34 (6.37)	8 (7.55)	26 (6.07)			1 (0.83)	3 (1.83)	13 (10.92)	17 (12.98)			8 (4.26)	26 (7.51)		
Average duration of use				*χ*^2^ = 3.20	0.362					*χ*^2^ = 9.43	0.398			*χ*^2^ = 0.83	0.843
<15 min	227 (42.51)	45 (42.45)	182 (42.52)			53 (44.17)	64 (39.02)	48 (40.34)	62 (47.33)			79 (42.02)	148 (42.77)		
15–30 min	240 (44.94)	52 (49.06)	188 (43.93)			47 (39.17)	76 (46.34)	61 (51.26)	56 (42.75)			86 (45.74)	154 (44.51)		
30–60 min	48 (8.99)	5 (4.72)	43 (10.05)			14 (11.67)	19 (11.59)	7 (5.88)	8 (6.11)			18 (9.57)	30 (8.67)		
>60 min	19 (3.56)	4 (3.77)	15 (3.50)			6 (5.00)	5 (3.05)	3 (2.52)	5 (3.82)			5 (2.66)	14 (4.05)		

**p* < 0.05 (bolded), χ*^2^: Chi-square test.

This study used a 1-to-5 rating scale to analyze total scores on three dimensions related to Gen AI: knowledge, attitudes, and challenges. In the attitudes dimension, items Q8–Q11 were negatively worded and reverse-coded (i.e., positive scores 1–5 were converted to 5–1). Overall, the median knowledge score was 3.43 (interquartile range [IQR] 2.86–3.86), the median attitude score was 3.58 (IQR 3.33–3.83), and the median challenge score was 3.50 (IQR 3.17–3.92) (Table 6), indicating respondents had moderate to moderately high knowledge and attitudes, and relatively notable perceptions of challenges.

**Table 6:** Differences in Gen AI knowledge, attitude, and challenge average scores by gender, grade, and residence (*N* = 534).

Variables	*n* (%)	^*^Knowledge Score	Statistic	*p*	Attitudes score	Statistic	*p*	Challenges score	Statistic	*p*
Total	534 (100%)	3.43 (2.86, 3.86)			3.58 (3.33, 3.83)			3.50 (3.17, 3.92)		
Gender			*Z* = −1.64	0.102		*Z* = −0.68	0.495		*Z* = −1.93	0.054
Male	106 (19.85%)	3.57 (3.00, 3.86)			3.58 (3.42, 3.92)			3.50 (3.17, 4.00)		
Female	428 (80.15%)	3.43 (2.86, 3.86)			3.58 (3.33, 3.83)			3.42 (3.15, 3.83)		
Grade			*χ*^2^ = 3.93#	0.27		*χ*^2^ = 7.21#	0.066		*χ*^2^ = 0.26#	0.967
Freshman	120 (22.47%)	3.43 (2.82, 3.86)			3.58 (3.33, 3.75)			3.50 (3.17, 3.92)		
Sophomore	164 (30.71%)	3.36 (2.71, 3.75)			3.58 (3.31, 3.83)			3.42 (3.15, 3.92)		
Junior	119 (22.28%)	3.43 (2.86, 3.86)			3.58 (3.33, 3.92)			3.50 (3.17, 3.75)		
Senior	131 (24.53%)	3.57 (3.00, 3.93)			3.67 (3.50, 3.92)			3.42 (3.08, 3.92)		
Residence			*Z* = −0.17	0.863		*Z* = −1.38	0.168		*Z* = −2.77	0.006
Rural	188 (35.21%)	3.43 (2.86, 3.86)			3.58 (3.33, 3.92)			3.33 (3.08, 3.67)		
Urban	346 (64.79%)	3.43 (2.86, 3.86)			3.58 (3.33, 3.83)			3.50 (3.17, 3.92)		

* Reverse Coding: For items Q8 to Q11, negative items will have their scores reversed (1–5 becomes 5–1). M: Median, Q₁: 1st Quartile, Q₃: 3rd Quartile.

*Z*: Mann–Whitney test, #: Kruskal-Wallis test.

Since the score data were not normally distributed, medians and interquartile ranges were used for description, and group differences were analyzed using the Mann–Whitney U test (*Z* values) and the Kruskal-Wallis test (*χ*^2^ values). By gender, knowledge scores (male 3.57, female 3.43, *Z* = −1.64, *p* = 0.102), attitude scores (male 3.58, female 3.58, *Z* = −0.68, *p* = 0.495), and challenge scores (male 3.50, female 3.42, *Z* = −1.93, *p* = 0.054) showed no statistically significant differences. However, the challenge score *p*-value approached significance, suggesting males might perceive slightly greater challenges. Among the grades, knowledge (*χ*^2^ = 3.93, *p* = 0.27), attitudes (*χ*^2^ = 7.21, *p* = 0.066), and challenges (*χ*^2^ = 0.26, *p* = 0.967) showed no significant differences; the attitude score *p*-value was near significant, indicating possibly more positive attitudes among higher-grade students. Regarding place of residence, knowledge (*Z* = −0.17, *p* = 0.863) and attitudes (*Z* = −1.38, *p* = 0.168) did not differ significantly; however, challenge scores differed significantly (*Z* = −2.77, *p* = 0.006), indicating that urban students perceived greater challenges.

## 4 Discussion

This single-center cross-sectional study systematically examined the status of generative AI (Gen AI) use among undergraduate nursing students in western China. The median scores M (Q1, Q3) for knowledge, attitude, and perceived challenges were 3.43 (2.86, 3.86), 3.58 (3.33, 3.83), and 3.50 (3.17, 3.92), respectively. Based on the classification of low (1.00–2.49), moderate (2.50–3.49), and high (3.50–5.00), students demonstrated a moderate level of AI knowledge. At the same time, their attitudes and perceived challenges fell within the high range but were close to the lower boundary of that range. This suggests that students have a basic awareness of AI but a limited understanding of its deeper technical principles and practical applications. Their generally positive attitudes and relatively frequent engagement indicate a willingness to explore AI, yet skill gaps, technical barriers, and data security concerns may hinder the translation of behavioral intention into actual use.

Interpreted through the TAM, these findings highlight that systematically enhancing AI literacy, providing hands-on training, and reducing external barriers are essential for strengthening students’ confidence and competence in using AI tools. Implementing these strategies can facilitate the effective integration of generative AI into nursing education and clinical practice.

### 4.1 Analysis of the current use of Gen AI tools

The results of this study show that 57.86% of undergraduate nursing students reported frequent or consistent use of GenAItools. Among them, 94.76% accessed these tools via smartphones, 70.41% used two to three different tools, and 84.46% primarily used them to solve problems. Only 1.50% of students reported never having used GenAI tools, and 87.45% indicated that their daily usage duration was less than 30 min. The primary sources of information about these tools were social media (78.09%) and peer recommendations (71.35%). In terms of tool preferences, DeepSeek (72.10%) and Doubao (69.85%) were the most popular, while the usage rate of ChatGPT was relatively low at only 7.49%. In contrast, at the School of Nursing, Kent State University in Ohio, USA, ChatGPT was the most widely used tool, with 93% of students (*n* = 102) reporting its use (28). This disparity may be influenced by educational resources, regional constraints, internet accessibility, and cultural differences. Several factors may explain these usage patterns (24, 29–31): (1) the widespread availability of smartphones enables convenient access to AI tools anytime and anywhere; (2) students tend to use multiple tools to meet various learning needs; (3) academic tasks drive students to use AI to enhance learning efficiency; (4) informal channels such as social media and peer recommendations serve as the primary means of information acquisition; (5) limited institutional investment in AI education leads students to rely more on self-directed exploration; and (6) regional and internet restrictions affect the accessibility of some international tools, resulting in a lower usage rate of ChatGPT.

### 4.2 Analysis of the current knowledge of Gen AI

The median score for students on the knowledge dimension was 3.43 (IQR 2.86–3.86), indicating a moderately low level of understanding of GenAI. Students possess basic conceptual knowledge but lack in-depth comprehension. Specifically, 55.36% agreed or strongly agreed that they “know what artificial intelligence is,” suggesting a relatively common foundational awareness. However, only 40.06% reported understanding machine learning or deep learning, reflecting limited familiarity with more technical concepts. About 56.08% indicated familiarity with the application of AI in the medical field, which may be influenced by general media exposure. In contrast, only 47.56% claimed to understand “current generative AI,” highlighting a limited grasp of emerging trends and technologies. Several factors may explain these disparities in knowledge (17, 19, 32, 33): (1) The tertiary education system within nursing schools in western China lacks structured AI training, with students mainly relying on social media and peer communication for information acquisition; (2) Nursing curricula in these institutions do not sufficiently cover AI-related content, and educational updates are often delayed; (3) Students’ exposure to technology varies significantly due to differences in personal interest, resource availability, and geographical constraints; (4) A lack of hands-on experience limits both comprehension and practical application; (5) Students exhibit weak capabilities in interacting with AI, as only 24.00% reported being able to formulate practical questions, indicating a need for improvement in critical thinking and information extraction skills.

To enhance students’ AI knowledge, it is crucial to strengthen structured AI education within the nursing school context, incorporate practical training components, and boost students’ confidence and competence in utilizing AI tools. Particular emphasis should be placed on cultivating the ability to formulate key questions, evaluate AI-generated outputs, and integrate AI applications into clinical contexts (34). To advance generative AI literacy in nursing education, some scholars have already called for nurse educators to take the lead in integrating generative AI concepts and tools into their curricula (35).

### 4.3 Analysis of current attitudes toward Gen AI

The median score for the attitude dimension was 3.58 (IQR 3.33–3.83), indicating that students generally hold a positive attitude toward GenAI. Specifically, 74% of students believed that AI is necessary for the development of healthcare, and 61% expected it to become a key component of future healthcare systems; 73.78% agreed that AI could improve the quality and efficiency of nursing care, and 71.16% supported recognizing it as part of technological advancement; 66.29% acknowledged the importance of AI in nursing, 60.67% agreed that budget allocation should support AI development, and 72.47% expressed a desire to see more AI-related content integrated into their curriculum. Despite the overall optimism, specific concerns persist: 28.00% worried that AI might replace nurses, 40.00% were concerned about misdiagnosis or overreliance on AI, 43.07% believed AI could increase workload, and 16.48% were worried that AI might undermine the humanistic aspects of care. These positive attitudes may be attributed to several factors (23, 36–40): (1) early exposure to smart devices has strengthened students’ confidence in AI applications; (2) AI is perceived to improve job competitiveness and students tend to be open to innovation; (3) AI tools are seen as helpful in saving time, enhancing academic performance and learning efficiency, and reducing repetitive tasks; (4) the digital environment and school context have encouraged awareness and interest in AI; (5) exploratory learning at the undergraduate level fosters acceptance of new technologies, with GenAI supporting interdisciplinary learning; (6) Innovative approaches like AI-supported flipped classrooms promote active learning, help students use AI effectively, and anticipate its role in personalized healthcare and data-driven decision-making. A recent mixed-methods study involving 33 Web Design and Coding students demonstrated that such classrooms enhanced AI literacy, increased motivation, fostered personalized and interactive learning, and improved critical thinking and problem-solving skills (41).

### 4.4 Analysis of challenges in using Gen AI

The median score for the challenge dimension was 3.50 (IQR 3.17–3.92), indicating a moderately high level of perceived difficulty among students when using GenAI. In terms of technology and usability, 32.27% of students reported that the interface was complex, and 31.27% experienced issues with data synchronization, indicating a need for improvement in human-computer interaction design. Concerns around data security and ethics were particularly significant, with 64.60% of respondents worried about data breaches and 61.43% concerned about copyright issues. Additionally, 49.62% cited high usage costs, and 44.20% were worried about the rapid pace of technological updates, highlighting issues related to affordability and sustainability (42–44). These challenges are mainly attributed to: (1) poor interface and interaction design leading to operational complexity; (2) inadequate data security measures and lack of robust encryption and privacy mechanisms; (3) high costs and rapid iteration in AI development and maintenance; (4) a lack of systematic education on AI, with students relying primarily on informal sources for learning; and (5) resource and regional disparities resulting in inconsistent user experiences. To address these issues, future efforts should focus on improving the user-friendliness of AI systems, optimizing data synchronization mechanisms, establishing stricter data protection and ethical standards, developing cost-effective and sustainable AI solutions, and promoting interdisciplinary collaboration to enhance the quality of AI integration in nursing education (45–47).

### 4.5 Analysis of group differences in the use of Gen AI

This study found that, while differences in Gen AI tool usage among student subgroups were primarily insignificant, some patterns emerged. Male students reported slightly higher rates of occasional AI tool use (31.27%) compared to females (29.25%), and a near-significant trend was observed, suggesting that males perceived slightly greater challenges than females (*Z* = −1.93, *p* = 0.054). This may indicate that male students are somewhat more cautious in using AI, possibly due to differences in learning style, technical experience, or confidence in AI technology. As students progressed through academic years, the frequency of AI tool use increased. For example, the proportion of first-year students who always used AI tools (19.25%) was lower than that of sophomores (22.64%), and the proportion of juniors who often used AI tools (33.96%) was higher than that of first-year students (37.64%). A comparison between urban and rural students showed that urban students reported slightly higher frequent usage (38.44% vs. 36.17%), and also perceived greater challenges (*Z* = −2.77, *p* = 0.006), possibly due to their higher expectations and sensitivity toward AI tools. These disparities may be attributed to factors such as (36, 48, 49): (1) disparities in educational resources and internet infrastructure, (2) increased exposure to AI-related content with academic progression, (3) urban students’ heightened expectations and sensitivity to new technologies, and (4) gender-based differences in attitudes, learning styles, or technical confidence regarding AI. Therefore, future educational interventions should consider these subgroup characteristics and implement targeted strategies to promote equity in AI education, thereby enhancing the technical literacy and application capabilities of students from diverse backgrounds (50–53).

## 5 Conclusion

This study provides a comprehensive assessment of the current status, attitudes, and challenges associated with the use of Gen AI among undergraduate nursing students in western China. The findings reveal that while students exhibit moderate knowledge and generally positive attitudes toward Gen AI, significant challenges remain, including concerns about data privacy, tool reliability, and the potential impact on critical thinking skills. The widespread adoption of AI tools, particularly through smartphones, underscores their integration into academic routines; however, the reliance on informal sources for AI-related information highlights gaps in structured education. Targeted interventions, such as incorporating AI literacy into curricula and addressing ethical and usability concerns, are essential to optimize the benefits of AI in nursing education.

Importantly, this study also developed and preliminarily validated a structured GAILQ-NS, which provides a valuable tool for assessing AI literacy in nursing students. Future applications of this instrument in diverse contexts will further contribute to the evaluation and improvement of AI education. Furthermore, future research should investigate the long-term implications and regional variations to inform further the responsible integration of AI technologies in healthcare education and practice.

## 6 Limitations

Several limitations of this study should be acknowledged. First, the sample consisted of undergraduate nursing students at Zunyi Medical University in western China, which may limit the generalizability of the findings to other regions or educational contexts. Second, data collection relied on self-reported questionnaires, which were subject to recall bias and subjective interpretation. Third, the cross-sectional design restricts the ability to infer causal relationships. Additionally, although the GAILQ-NS underwent preliminary validation, its reliability and validity require further confirmation in broader populations. Future studies should consider employing more diverse samples and combining quantitative and qualitative methods to provide a more comprehensive assessment of the application of Gen AI in nursing education.

## Data Availability

The original contributions presented in the study are included in the article/supplementary material, further inquiries can be directed to the corresponding authors.

## Footnotes

^1^ https://www.calculator.net/sample-size-calculator.html
